# The comprehensive updated regulatory network of *Escherichia coli *K-12

**DOI:** 10.1186/1471-2105-7-5

**Published:** 2006-01-06

**Authors:** Heladia Salgado, Alberto Santos-Zavaleta, Socorro Gama-Castro, Martín Peralta-Gil, Mónica I Peñaloza-Spínola, Agustino Martínez-Antonio, Peter D Karp, Julio Collado-Vides

**Affiliations:** 1 Bioinformatics Research Group, SRI International, 333 Ravenswood Ave EK207, Menlo Park CA 94025 USA.; 2 Program of Computational Genomics, Centro de Ciencias Genómicas, Universidad Nacional Autónoma de México. A.P. 565-A Cuernavaca, Morelos 62100, Mexico.

## Abstract

**Background:**

*Escherichia coli *is the model organism for which our knowledge of its regulatory network is the most extensive. Over the last few years, our project has been collecting and curating the literature concerning E. coli transcription initiation and operons, providing in both the RegulonDB and EcoCyc databases the largest electronically encoded network available. A paper published recently by Ma et al. (2004) showed several differences in the versions of the network present in these two databases.

Discrepancies have been corrected, annotations from this and other groups (Shen-Orr et al., 2002) have been added, making the RegulonDB and EcoCyc databases the largest comprehensive and constantly curated regulatory network of *E. coli *K-12.

**Results:**

Several groups have been using these curated data as part of their bioinformatics and systems biology projects, in combination with external data obtained from other sources, thus enlarging the dataset initially obtained from either RegulonDB or EcoCyc of the *E. coli *K12 regulatory network. We kindly obtained from the groups of Uri Alon and Hong-Wu Ma the interactions they have added to enrich their public versions of the *E. coli *regulatory network. These were used to search for original references and curate them with the same standards we use regularly, adding in several cases the original references (instead of reviews or missing references), as well as adding the corresponding experimental evidence codes. We also corrected all discrepancies in the two databases available as explained below.

**Conclusion:**

One hundred and fifty new interactions have been added to our databases as a result of this specific curation effort, in addition to those added as a result of our continuous curation work. RegulonDB gene names are now based on those of EcoCyc to avoid confusion due to gene names and synonyms, and the public releases of RegulonDB and EcoCyc are henceforth synchronized to avoid confusion due to different versions. Public flat files are available providing direct access to the regulatory network interactions thus avoiding errors due to differences in database modelling and representation. The regulatory network available in RegulonDB and EcoCyc is the most comprehensive and regularly updated electronically-encoded regulatory network of *E. coli *K-12.

## Background

*Escherichia coli *is a reference organism because of the rich knowledge of its biology. The group of Collado-Vides has been curating regulation of transcription initiation and operon organization during the last ten years [1-3]. This effort feeds two databases, RegulonDB and EcoCyc [4,5]. This information is freely and openly available, and has been used by several groups to perform different types of analyses, in bioinformatics and systems biology.

For instance, this dataset was used a few years ago by the group of Uri Alon to identify statistically over-represented topological motifs of gene-regulatory relationships within the network [6]. The notion of statistically significant motifs proposed therein has been widely used in subsequent analyses of networks. More recently, a team led by An-Ping Zeng in Braunschweig has analyzed topological properties of this network [7]. These two groups have enlarged this dataset with their own addition of interactions.

Additional uses of this dataset include novel or expanded computational methods of promoters; operons [8-10]; microarray analyses [11,12]; and chip-chip experiments [13]; formal models of transcriptional processes [14]; metabolic and regulatory network reconstruction [15]; and experimental studies [16,17], among others.

## Results

In order to avoid a multiplication of slightly different versions of the *E. coli *regulatory network, we have integrated the interactions from the Alon and Zeng groups – data generously provided by them – and have flat files publicly available that contain in a direct and simple way the set of regulatory interactions either at the gene or at the protein level.

Ma et al have extracted from EcoCyc and from RegulonDB the regulatory network of interactions and the transcriptional links of sigma factors and their regulated genes. They describe in their Figure 1 an important number of differences in the network obtained from each database. There are several reasons for these differences, summarized below, but an important one derives from the different ways of encoding the interactions in the schemas of EcoCyc and RegulonDB. Furthermore, they used the two versions available then, version 8.0 of EcoCyc and version 4.0 of RegulonDB which were not synchronized and therefore had a different content. As mentioned below, since version 9.0 of EcoCyc and 4.4 of RegulonDB, the public versions of both databases are synchronized to prevent these problems. Version 9.6 of EcoCyc will correspond to version 5.0 of RegulonDB.

In the following we describe the specific differences and curation involved, and in the last sub-section we describe the final files available with the updated network as well as those apparent interactions that were not incorporated.

### Unified use of gene names and synonyms

Some of the differences in the network observed by Ma et al derive from discrepancies in gene names and in interactions when comparing each database. Differences in gene content are mostly due to the use of different names and synonyms in each database. They have been corrected, and we now make uniform use of gene names and synonyms, mostly incorporating the curated gene annotations of EcoCyc into RegulonDB in future releases.

Ma et al found 370 interactions present in RegulonDB and absent in EcoCyc and 336 present in EcoCyc and absent in RegulonDB. As summarized in Table 2, after eliminating repetitions in both databases that derive from the existence of heterodimer proteins (i.e. IHF encoded by himA and himD genes, rcsA and rscB genes that encode for protein RcsB), separating the set of sigma factor interactions with their corresponding promoters as a different type of interactions, as well as those that are already present in both databases as curation continued, we were left with 69 interactions unique to RegulonDB and 77 unique to EcoCyc.

**Table 2:** Summary of the curation made to the Alon and Ma data.

	***RegulonDB***	***EcoCyc***
Numbers from Fig 1 in Ma et al. unique to each database	370	336
Repetitions coming from heterodimer regulators (hupB, ihfB, rcsB)	29	19
Sigma factor regulated genes	88	8
Curated and present in both databases	184	294
Final discrepancies – different synonyms, etc (see text)	69	77

Out of the 69 considered unique to RegulonDB, 24 interactions are in fact present in both databases but they were missed by Ma et al. because the gene names are different. Some gene names in one database are synonyms in the other. We have unified the names in both databases with the first one in the following list of pairs being the current name, followed by its synonym: atsC-argM, mngA-hrsA, mngR-farR, mazF-chpA, mazE-chpR, rrnA-rrsA, rrnC-rrsC, rrnD-rrsD, rrnE-rrsE, rrnG-rrsG, rrnH-rrsH, csiR-gabC, glnG-ntrC, astC-argM, nfnB-nfsB, alsR-rpiR.

Out of the interactions reported as unique to EcoCyc, six were in fact present in both databases but with different regulated gene names, although present as synonyms. The pair of gene names and synonyms is the following: mlc-dgsA, icdA-icd, lpdA-lpd. Since mlc encodes a TF, and it regulates 3 other genes, its name change affects 3 interactions. Furthermore, lpdA is regulated by two different Tfs (ArcA and Fis) thus again one gene name affects two TF-gene interactions.

### Specific corrections

Ma reports interactions present in one database and not in the other one. However, in our analyses they are absent in both. There are 17 such interactions with no promoter associated, therefore they do not belong to any known TU, and we do not consider them as validated interactions.

Finally, 28 specific regulator-regulated gene interactions, affecting 27 regulated genes, (two interactions affect *gcvB*), implied correcting only 15 interactions as encoded in the databases because four operons (sufABCDSE, galETKM, rtcBA and guaBA) account for 14 TF-regulated gene interactions, in addition to 11 interactions of genes transcribed monocistronically.

77 interactions are reported as unique to EcoCyc. These involve 19 proteins affecting 61 genes. In addition to those due to different gene names mentioned above, there are 8 interactions involving glnL, which encodes a sensor protein NtrB. In fact all these genes are regulated by NtrC, encoded by glnG. These are interactions wrongly derived from EcoCyc that were never present in the databases and are not present in the current website.

The knowledge in the databases is sometimes incomplete, such as in some cases, annotated promoters, for which there is no clear evidence of the extent of the TU they transcribe. In EcoCyc such promoters are linked with the downstream TU, even if there is no experimental evidence for the existence of the TU. This generates differences in the number of interactions, accounting for 16 additional interactions present in EcoCyc and absent in RegulonDB. It is up to the criteria of the modeler of the network to assume that such likely interactions exist, or from a more conservative approach, to eliminate them. In the website mentioned below, they are not included.

18 interactions were in fact corrected before, first deleted in RegulonDB, but present in version 8.0 of EcoCyc and deleted in subsequent releases.

We corrected 11 operons which involve 29 TF-regulated gene interactions. These affect a total of 23 genes -again because some of them are regulated by more than one TF-. 17 of the 29 specific interactions are grouped in four operons: pdhR-aceEF-lpdA, deoABCD, glpTQ and fimAICDFGH.

### Final datasets of the regulatory network and of transcription by different types of RNAPs based on their sigma factors

The RegulonDB and EcoCyc data web pages will be updated systematically as part of the periodic releases of EcoCyc and RegulonDB. Both the team of Uri Alon and the team of An-Ping Zeng have agreed to link to our urls from their respective public sites.

The datasets produced as a result of the curation described above, are the following:

#### Interactions available both in EcoCyc and RegulonDB

File 1 RegulonDB-EcoCyc interactions, contains the set of regulatory interactions at the level of transcription initiation. Each line describes the name of the transcription factor (TF), the regulatory gene, the name of the regulated gene, and the corresponding function (activator or repressor). In the case of heterodimers, we kept them in one line, with for instance, IHF, followed by himA/himD genes. Thus, the total counting corresponds to the number of proteins affecting the expression of individual genes. Note that these are exclusively regulatory interactions affecting the initiation of transcription.

File 2 Gene Sets Transcribed by common Sigma Factors contains the set of seven sigma factors present in *E*. *coli*, and their corresponding transcribed genes. These are not regulatory interactions, but sets of genes transcribed by the same RNA polymerase holoenzyme.

#### Interactions not found in *E. coli *K-12

File 3 and File 4, have those interactions for which we found no evidence and were not therefore added to the databases. File 3 has 19 interactions from Alon for which we found no published evidence. File 4 has 31 interactions from Ma et al.

12 interactions that are not of transcriptional regulation (pending curation), 10 based on microarray experiments which we cannot know if are direct or indirect, and 9 for which we found no reference of experimental evidence. Table 1 summarizes the literature revision performed to what initially were 346 interactions of Alon and 93 of Zeng that were absent in RegulonDB and EcoCyc. As mentioned before, after curation of these two datasets we have 150 new interactions that have been curated.

**Table 1:** Summary of differences comparing with the regulatory interactions from Ma.

	Alon	Ma
New interactions reported by Alon and Ma	**346**	**93**
		
New Regulatory interactions added in RegulonDB and EcoCyc	69	27
New Sigma Factor Regulatory interactions added in RegulonDB and EcoCyc	32	22
		
Regulatory interactions already included in the last release	130	13
Sigma factor regulatory interactions already included in the last release	93	
Regulatory interactions updated	3	
Other kinds of regulation		12
Interactions inferred from microarray data		10
Interactions not found in any literature source	19	9

### Access to the regulatory network

Access to more complete data on transcriptional regulation and operon organization can be found at RegulonDB [18] and EcoCyc [19] web sites. The public releases of both databases are now synchronized.

## Conclusion

As mentioned before, the data sets available from RegulonDB and EcoCyc web pages shall facilitate investigators to generate the full regulatory network of *E. coli*. The remaining few differences between the databases will disappear as curation of the data now occurs within EcoCyc and is immediately propagated to RegulonDB. We will extract from EcoCyc the annotations of genes and incorporate them for the genome annotation in RegulonDB. Furthermore, the releases of both databases have been synchronized, to avoid different versions of the data content. There will be a corresponding equivalent version of each database.

## Authors' contributions

HS carried out the data analysis, synchronized the regulatory network data sets and drafted and revised the manuscript. AS participated and coordinated the curation of data, SG, MP and MIP participated in the curation and analysis of data, AM participated at the beginning of the project in its coordination and drafted a first manuscript, PDK was involved in drafting the manuscript and revising it critically, JC conceived the study, and drafted and revised the manuscript.
